# Supplementary figures and images for: The role of heterochronic gene expression and regulatory architecture in early developmental divergence
Source: eLife. 2024 Aug 23;13:RP93062. doi: 10.7554/eLife.93062 (PMC11343563; doi:10.7554/eLife.93062)

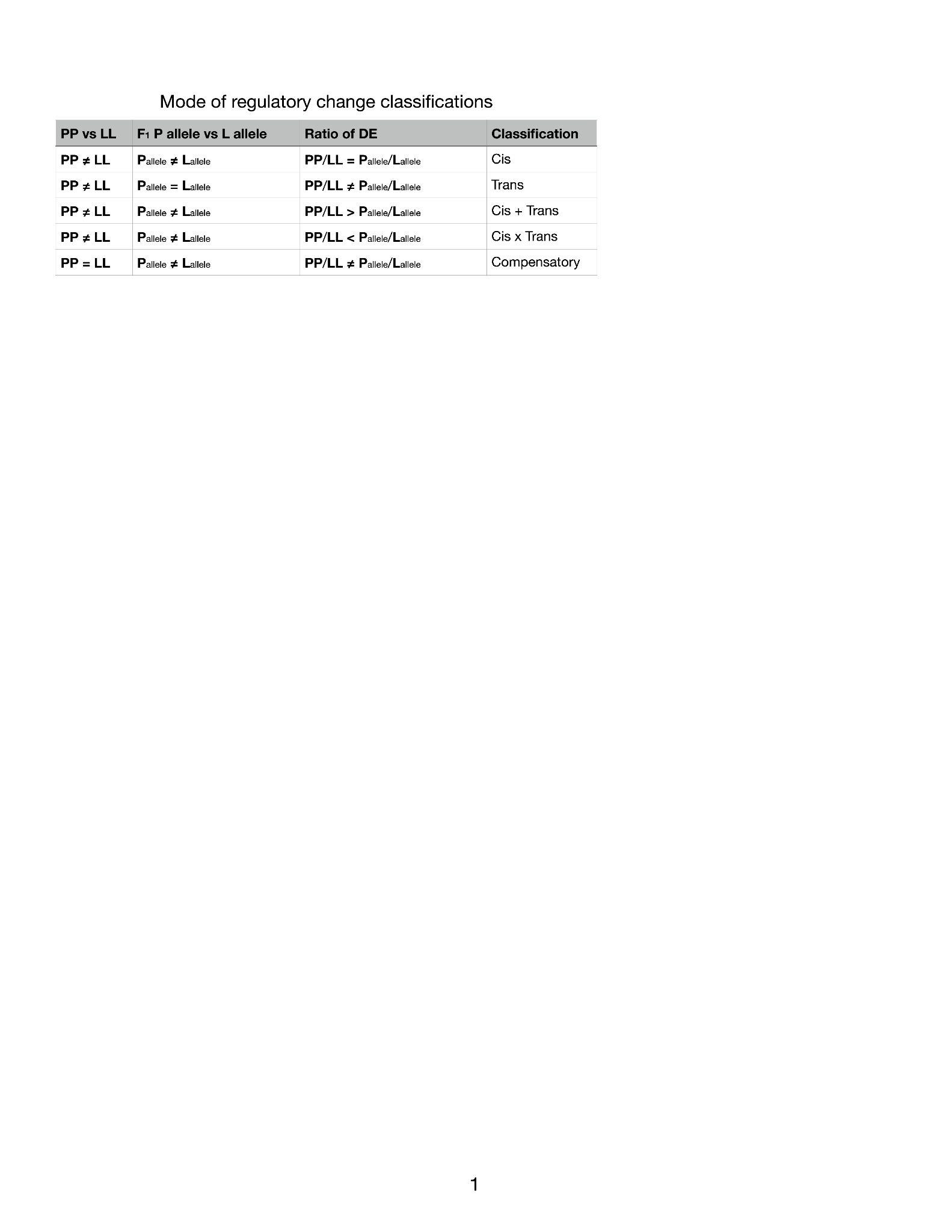

Supplement: Supplementary file 2. [file elife-93062-supp2.docx]
